# Interaction between γ-Hydroxybutyric Acid and Ethanol: A Review from Toxicokinetic and Toxicodynamic Perspectives

**DOI:** 10.3390/metabo13020180

**Published:** 2023-01-25

**Authors:** Suryun Jung, Mingyu Kim, Suji Kim, Sooyeun Lee

**Affiliations:** College of Pharmacy, Keimyung University, 1095 Dalgubeoldaero, Dalseo-gu, Daegu 42601, Republic of Korea

**Keywords:** gamma-hydroxybutyric acid, ethanol, drug abuse, toxicokinetics, toxicodynamics

## Abstract

Gamma-hydroxybutyric acid (GHB) is a potent, short-acting central nervous system depressant as well as an inhibitory neurotransmitter or neuromodulator derived from gamma-aminobutyric acid (GABA), a major inhibitory neurotransmitter. The sodium salt of GHB, sodium oxybate, has been used for the treatment of narcolepsy and cataplexy, whereas GHB was termed as a date rape drug or a club drug in the 1990s. Ethanol is the most co-ingested drug in acute GHB intoxication. In this review, the latest findings on the combined effects of GHB and ethanol are summarized from toxicokinetic and toxicodynamic perspectives. For this purpose, we mainly discussed the pharmacology and toxicology of GHB, GHB intoxication under alcohol consumption, clinical cases of the combined intoxication of GHB and ethanol, and previous studies on the toxicokinetic and toxicodynamic interactions between GHB and ethanol in humans, animals, and an in vitro model. The combined administration of GHB and ethanol enhanced sedation and cardiovascular dysfunction, probably by the additive action of GABA receptors, while toxicokinetic changes of GHB were not significant. The findings of this review will contribute to clinical and forensic interpretation related to GHB intoxication. Furthermore, this review highlights the significance of studies aiming to further understand the enhanced inhibitory effects of GHB induced by the co-ingestion of ethanol.

## 1. Introduction

Gamma-hydroxybutyrate (GHB) is an endogenous short fatty acid, acting as an inhibitory neurotransmitter or neuromodulator in mammalian brains. Endogenous GHB is produced during the metabolic process of gamma-aminobutyric acid (GABA), an inhibitory neurotransmitter that is mainly derived from glutamic acid in neurons, and is again metabolized to GABA. The sodium salt of GHB, sodium oxybate (Xyrem^®^), was approved by the U.S. Food and Drug Administration as a central nervous system depressant for narcolepsy and cataplexy in 2002, while GHB was termed as a date rape drug or a club drug in the 1990s [1,2]. Subsequently, the precursors of GHB, 1,4-butanediol (1,4-BD) and gamma-butyrolactone (GBL), emerged as alternatives for illegal use. Exogenous GBL is converted into GHB by a lactonase that is present in the liver and serum but not in the brain, and exogenous 1,4-BD is oxidized via alcohol dehydrogenase (ADH) into gamma-hydroxybutyraldehyde, which is further metabolized by aldehyde dehydrogenase (ALDH) to GHB. Thus, both agents have the same psychoactive effects as GHB [3,4,5].

Alcohol, a neuro-inhibitor, is one of the most consumed and easily accessible psychoactive substances globally. In particular, certain alcohol consumption patterns such as binge drinking and drink spiking are serious social and public health issues and are often involved in crimes worldwide [6]. Ethanol is the most co-ingested drug, probably augmenting pleasure and euphoric effects of other psychoactive drugs, represented in emergency department visits of acute recreational drug intoxication in European countries [7,8]. The mechanism of action of acute ethanol exposure is complicated and involves multiple steps, i.e., directly inhibiting ADH as well as acting on a number of neurotransmitter receptors (e.g., GABA_A_, glycine, and glutamate receptors), ion channels (e.g., large-conductance Ca^2+^-activated K^+^ channel and G-protein-coupled inwardly rectifying K^+^ channel), and other non-ion-channel targets, including intracellular signaling molecules such as protein kinase C and adenylate cyclase in the brain [9]. Ethanol is metabolized by ADH, ALDH, and cytochrome P450 enzymes such as CYP2E1, CYP1A2, and CYP3A4 which are also involved in the metabolisms of other drugs. Acute alcohol exposure affects the neuro-stimulant or inhibitory effects of other psychoactive drugs. In particular, high doses of alcohol, which strongly induce inhibitory effects, reduced the stimulant effects or enhanced the inhibitory effects of concomitant drugs [8,10]. It was also reported that low doses of alcohol induced neuro-stimulant effects on behavioral changes caused by the administration of GHB [11]. Therefore, the combined effects of GHB and ethanol could be diverse; however, mechanisms underlying the GHB and ethanol interactions are not yet fully understood.

## 2. Search Methods and Results

In this review, the latest findings on the combined effects of GHB and ethanol are summarized from toxicokinetic and toxicodynamic perspectives. For this purpose, we mainly discussed the pharmacology and toxicology of GHB, GHB intoxication under alcohol consumption, clinical cases of the combined intoxication of GHB and ethanol, and previous studies on the toxicokinetic and toxicodynamic interactions between GHB and ethanol in humans and animals. We reviewed research papers published from January 1980 to March 2022 and listed them in PubMed. The search keywords were [“γ-hydroxybutyric acid”, “γ-hydroxybutyrate”, “GHB”, “1,4-butanediol”, “1,4-BD”, “γ-butyrolactone”, “GBL”] and [“Ethanol” or “Alcohol”].

**Figure 1:**
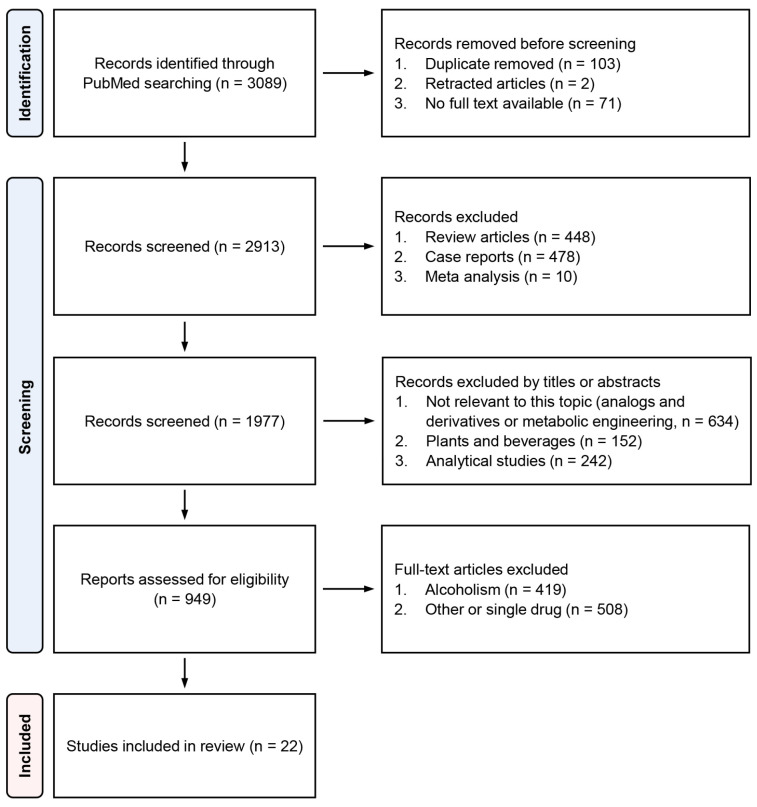
Flow diagram of paper selection steps.

We found a total of 3089 papers with the aforementioned search keywords, and 22 of these papers, including three clinical case studies and human studies, 15 animal studies, and one in vitro study, were selected for the systematic review [11,12,13,14,15,16,17,18,19,20,21,22,23,24,25,26,27,28,29,30,31,32]. The flowchart with details of paper selection steps is presented in Figure 1.

## 3. Pharmacology and Toxicology of GHB

GHB includes endogenous GHB synthesized in neurons and exogenous GHB acting through the blood–brain barrier (BBB) after ingestion. The physiological effects of GHB are mediated through binding to the GHB-specific receptor, identified as the α_4_β_1_δ subtype of the GABA_A_ receptor, which is one of the high-affinity targets activated by nano-micromolar concentrations of GHB. The pharmacological and toxicological effects of GHB are mainly attributed to its action at the GABA_B_ receptor, which is one of the low-affinity targets activated by millimolar concentrations of GHB [33]. Moreover, animal studies demonstrated that GHB administration up- or downregulates the extracellular concentrations of GABA, glutamic acid, and dopamine in the brain depending on drug doses or the specific action regions of the brain [33,34,35,36].

GHB was first synthesized as an anesthetic in 1964 [37] but had not been commercialized because of its severe side effects, such as vomiting and convulsions. In the late 1980s, GHB was widely used by bodybuilders as a performance-enhancing agent for anabolic steroids and became readily available as an over-the-counter product in health food stores, gyms, and the Internet. Subsequently, since GHB was shown to elicit euphoria and sexual arousal, it was increasingly misused in dance clubs and ‘rave’ parties [38]. In 2000, GHB was classified as a Schedule I substance because it was frequently abused in sexual assaults and, when taken in excess, caused coma, bradycardia, agitation, hypotension, hypothermia, respiratory depression, and, in extreme cases, death [5,39]. However, increased GHB production is beneficial for protecting central and peripheral tissues during hypoxia, ischemia, or excessive metabolic demand [40]. GHB has a neuroprotective action against apoptosis and neuronal death caused by oxidative stress [41,42,43]. In addition, GHB interferes with sleep latency, promotes deep slow-wave non-rapid eye movement sleep [44,45], and has sedative effects [46,47]. GHB was also found to be effective in the treatment of narcolepsy [48] and alcohol and heroin dependence [49].

## 4. GHB Intoxication in Crimes under the Influence of Drugs

The acute intoxication of GHB, an illegal drug misused in crimes under the influence of drugs owing to its neuro-inhibitory property, has greatly increased recently and threatens public health report [50,51]. GHB is colorless, tasteless, and short-acting; thus, it can be surreptitiously administered to a victim’s drink, such as alcohol, to incapacitate victims and induce memory loss in crimes, such as robberies, sexual assaults, and fraudulent gambling. However, due to its short half-life (30–50 min), low urine excretion rate (less than 2%), endogenous presence in both the brain and other peripheral tissues and fluids, and post-mortem production, it is difficult to prove previous GHB administration through quantification of GHB in a victim’s biological specimens such as blood and urine [33,52,53]. To overcome this limitation, many previous studies have focused on the toxicokinetic evaluation of GHB to establish a detectable sampling time [54,55,56], reference value setup based on endogenous GHB concentrations to interpret the quantitative results [57,58] and biomarker discovery for GHB exposure by metabolomics [59,60].

A combination of GHB and alcohol is frequent in crimes, and acute alcohol intake is known to cause toxicokinetic and toxicodynamic perturbation on concomitant psychiatric drugs [61]. Both GHB and ethanol could affect the common neuronal systems such as the GABAergic system. Moreover, it was previously reported that acute alcohol exposure alters the concentrations of dopamine, glutamic acid, and GABA in rats’ brains [62,63]. GHB is metabolized to 2,4- or 3,4-dihydroxybutyric acid or succinic acid which goes through the Krebs cycle [64,65] and then rapidly eliminated from the body in the form of carbon dioxide and water. GHB was detected only within 6 h in the blood and 12 h in urine after a single exposure to 60 or 50 mg/kg of GHB [16,66,67]. Ethanol is first oxidized to acetaldehyde and then to acetate and is finally processed by the Krebs cycle. It is eliminated approximately 5 h after consumption stops, and ethanol oxidation is the largest carbon source for energy metabolism during this period [68]. The effect of ethanol on the toxicokinetic, as well as toxicodynamic, characteristics of GHB should be addressed. Further in-depth studies on the combined effect of GHB and ethanol, based on a variety of conditions in animal and clinical studies, are necessary.

## 5. Clinical Case Studies of GHB/GBL and Ethanol-Related Intoxication

Numerous clinical case studies have reported that GHB intoxication is the main cause of hospitalizations related to club drug use [69,70,71,72,73,74]. In European countries, hospitalization rates were significantly higher in patients intoxicated with GHB than in those intoxicated with heroin [3]. However, many cases of GHB intoxication are easily misdiagnosed because of unfamiliar symptoms and confusion with ethanol poisoning or other sedatives [75]. The most common symptom of GHB intoxication was central nervous system depression associated with bradycardia, hypotension, and hypothermia, as well as other additional symptoms such as aggression, exhaustion, memory loss, vomiting, drowsiness, apnea, seizures, suffocation, and coma [76,77]. Most of these symptoms disappear within 4–8 h owing to the short clearance time of GHB and its precursors and metabolites [15]. In the case of GHB toxicity, some of the reported symptoms may be related to multidrug use, and the use of ethanol in combination was more dangerous than the use of GHB alone [14]. Only a few clinical studies have reported details of the case characteristics of co-ingestion of GHB and alcohol (Table 1). A study of drug-related problems in patients who visited emergency departments within the Euro-DEN network over a 12-month period found that 71.7% of patients consumed GHB with other abusive substances, among which 50% consumed GHB with alcohol [12]. Similarly, among patients with GHB poisoning who visited emergency departments in 14 European countries over a period of 39 months, 426 patients reported ethanol consumption with GHB or GBL [13]. Patients with GHB intoxication, with or without co-ingestion of alcohol or other drugs, commonly showed altered behavior, reduced consciousness, anxiety, agitation or aggressiveness, bradycardia, hypotension, and hypothermia. The patients co-ingesting GHB and ethanol showed a higher frequency of vomiting, cardiovascular symptoms, decreased consciousness, and agitation than those ingesting GHB alone. In particular, a higher dose of GHB enhanced agitation or aggressive behavior [12,13,14]. Table 1 notes that ethanol is the most common agent among other psychoactive substances used in combination with GHB and exacerbates toxicity caused by the administration of GHB.

## 6. Toxicokinetic and Toxicodynamic Interactions between GHB and Ethanol in Humans

Table 2 summarizes toxicokinetic and toxicodynamic studies in healthy adults. Thai et al. reported that the mixed intake of GHB (50 mg/kg) with ethanol (0.6 g/kg) in 16 healthy adults increased maximal concentration (C_max_) by 16% and elimination half-life by 29%, compared with GHB ingestion alone, but there was no statistically significant difference. Ethanol has the potential to influence the bioavailability or clearance rate of GHB [15]. In another study by Haller et al. for the same clinical trial design, urine GHB concentrations were lower in the first 3 h following the co-ingestion of ethanol and GHB. However, co-ingestion of ethanol did not significantly affect renal clearance of GHB [16]. These results imply that it is necessary to investigate the toxicokinetic interaction between GHB and ethanol for more diverse clinical trial designs employing various dosages of GHB and ethanol. In contrast, toxicodynamic interactions between GHB and ethanol were clearly shown. The co-administration of ethanol significantly reduced oxygen saturation as well as systolic and diastolic blood pressure and significantly increased the heart rate, compared with GHB ingestion alone. Moreover, higher frequencies of abnormal symptoms such as hypotension and vomiting were observed upon co-ingestion of ethanol and GHB [15]. The toxicokinetic and toxicodynamic interactions of differently formulated GHB and ethanol were also studied. When a single dose of a solid immediate-release formulation of sodium oxybate and ethanol were co-administered in 24 healthy adults, no toxicokinetic interaction was observed. However, alertness and stimulation were significantly increased and sedation was decreased within 60 min after the co-administration [17]. As shown in Table 2, combined exposure to ethanol induced significant changes to the toxicodynamics of GHB, while minor toxicokinetic changes were observed. However, it should be noted that only the therapeutic doses of GHB were used in all the three human studies.

## 7. Toxicokinetic and Toxicodynamic Interactions between GHB and Ethanol in Animals

Table 3 summarizes animal studies on toxicokinetic and toxicodynamic changes in GHB following co-administration of ethanol. In a previous study, behavioral changes following GHB and ethanol co-administration were investigated using the functional observational battery (FOB) [11]. FOB is a noninvasive procedure designed to detect and quantify gross functional deficits in young adult rats due to chemical exposure and is widely utilized as a neurobehavioral assessment tool to describe various behavioral and activity-related parameters in the rat strain [78]. Van Sassenbroeck et al. measured the whisker reflex (WR), startle reflex (SR), righting reflex (RR), and tail clamp reaction (TC) as FOB. This toxicodynamic interaction was studied using isobolographs and an interaction model in rats receiving a combination of steady state concentrations of ethanol (1000–3000 μg/mL) and GHB (200–1400 μg/mL). The results of this study showed that following GHB and ethanol co-administration, the effective concentration of GHB causing 50% of the maximal response (EC_50_) decreased in three reactions except WR. Additionally, RR showed a synergistic effect on GHB EC_50_ at higher ethanol concentrations (>2000 μg/mL) and additivity at lower ethanol concentrations. SR showed an antagonistic effect at ethanol concentrations of <1000 µg/mL and showed additivity at higher ethanol concentrations. The TC reaction was antagonistic at all ethanol concentrations. In addition, co-administration of 300 mg/kg GBL and 3 g/kg ethanol intraperitoneally significantly increased the sleep time compared with the administration of GBL alone (GBL, 66 ± 4 min; ethanol, 231 ± 9 min; GBL/ethanol, 389 ± 6 min) [11]. Cook et al. reported that the co-administration of GHB and ethanol significantly reduced locomotor activity and had a significant effect on the results of FOB compared to the administration of GHB alone. The co-administration of GHB and ethanol increased acute depressive behavioral responses by impairing RR and inverted screen performance (SIP), increasing hindlimb splay (HS), and decreasing forelimb grip strength (FGS) and body temperature [18]. This is similar to the results of another previous study where the co-administration of GHB and ethanol showed increased sedative effects [19]. However, other effects on the central nervous system observed in these two studies were inconsistent. In the former study, the co-administration of GHB (0.1–0.3 g/kg) and ethanol (3–4 g/kg) significantly reduced body temperature compared to the administration of GHB alone [18]. However, no difference in respiratory depression after co-administration of GHB and ethanol was observed in the latter study, despite the difference in the dosage of the drug (GHB, 1.5 g/kg) [19].

Mechanistic studies were conducted to elucidate the toxicological effects of GHB and ethanol. GHB-induced physiological and behavioral changes are caused by interactions with GHB-specific receptors, the GABA_A_ receptor, and GABA_B_ receptors in various regions of the brain [79,80,81]. It has been previously suggested that GHB is a substrate of the monocarboxylate transporter (MCT) family (SLC16A) [82,83] and sodium-binding MCT (SMCT) family (SLC5A) [84]. MCT1, a ubiquitous protein encoded by the human SLC16A1 gene, is expressed in the BBB and plays an important role in transporting substrates into and out of the brain [85]. MCT mediates renal reuptake of GHB [82,86], and GABA_A_ and GABA_B_ receptors mainly regulate the sedative effects of ethanol and GHB, respectively [87,88,89]. As shown in Table 3, Rodriguez-Cruz and Morris reported that MCT1 inhibitors (AR-C155858, AZD-3965) significantly reduced respiratory depression and sedative effects caused by the co-administration of GHB and ethanol and decreased the brain concentration and brain-to-plasma concentration ratio of GHB at return of righting reflex (RRR) [19]. Morse and Morris investigated the effects of the MCT inhibitor (L-lactate), GABA_A_ receptor antagonist (bicuculline), and GABA_B_ receptor antagonists (SGS742, SCH50911) on the toxicological interactions of GHB, and ethanol L-lactate significantly reduced sleep time, mortality, and brain-to-plasma ratio of GHB at RRR following the co-ingestion of GHB and ethanol. However, there was no significant difference in the brain’s GHB concentrations at the measured time point. Additionally, the administration of bicuculline did not affect respiratory depression following co-administration of GHB and ethanol while that of SCH50911 decreased the respiratory rate and completely prevented death from the administration of high concentrations of ethanol (0.3–0.4%) [20]. These previous studies demonstrated that the combined administration of GHB and ethanol increased inhibitory effects, but MCT1 inhibition could alleviate toxicity by inhibiting GHB brain uptake [19,20]. The inhibition of MCT has been paid attention to as a potential treatment strategy for GHB intoxication [90,91,92,93].

Fung et al. reported that 1,4-BD showed a more potent CNS inhibitory effect than GHB. Nevertheless, it was observed that the total duration of loss of righting reflex (LRR) due to 1,4-BD showed a tendency to decrease when administered with ethanol, while that due to GHB increased significantly [21]. Another study demonstrated that there were no significant differences in reinforcing effects and demand functions between ethanol administration alone and the co-administration of ethanol and GHB in the self-administration experiment in Rhesus monkeys [22]. Hicks and Varner reported that 1,4-BD is potentially more dangerous because it is approximately 10 times more potent as a cardiovascular stimulant than GHB when administered intragastrically [25]. Carai et al. also demonstrated that 1,4-BD traverses the BBB more quickly than GHB and has a stronger efficacy [24]. In both studies, ethanol reduced the risks associated with 1,4-BD [24,25].

Table 4 summarizes animal and in vitro studies on toxicokinetic and toxicodynamic changes in 1,4-BD following co-administration of ethanol. Since the inhibitory effects of 1,4-BD are mediated by its conversion to GHB by ADH [4], a major metabolic enzyme of ethanol, the co-administration of 1,4-BD with ethanol is considered to provoke significant metabolic interactions. The ethanol co-administration decreased the mean arterial pressure (MAP) and heart rate (HR) enhanced by 1,4-BD [23], inhibited the sedative effect [24,26], and reduced the mortality rate [22], which implies that competitive inhibition of ethanol in the conversion of 1,4-BD to GHB may alter the physiological effects attributed to 1,4-BD. Poldrugo and Snead reported that ethanol blocked the electroencephalogram activity of 1,4-BD when administered before 1,4-BD. Moreover, ethanol inhibited the elevation of GHB concentrations in the brain and liver of 1,4-BD-administered animals. From these results, the authors concluded that acute administration of ethanol recovered the inhibitory effects of 1,4-BD by blocking the degradation of 1,4-BD to its active metabolite GHB through competition for ADH [26]. When the ADH inhibitor, pyrazole, was administered to rats, the conversion rate of 1,4-BD to GHB in the liver was significantly reduced [27]. Treatment with another ADH inhibitor, fomepizole, in human-derived hepatocytes strongly inhibited the conversion of 1,4-BD to GHB [28]. On the other hand, Poldrugo et al. also reported that the co-administration of 1,4-BD and ethanol increased the mortality rate, compared with the administration of 1,4-BD alone, in rats and caused microscopic pathological changes in the liver and kidneys [29]. Further studies are necessary to investigate the toxicodynamic interactions between 1,4-BD and ethanol for more diverse animal study designs employing various dosages of 1,4-BD and ethanol.

The mechanism of the sedative effect of 1,4-BD was studied using ADH inhibitors (4-methylpyrazole, disulfiram, and ethanol), the GHB receptor antagonist (NCS-382), and GABA_B_ receptor antagonists (SCH 50911 and CGP 46381). 4-Methylpyrazole and ethanol completely prevented the sedative effect of 1,4-BD, and disulfiram partly blocked it. In addition, the sedative effects of 1,4-BD were antagonized by the GABA_B_ receptor antagonists, SCH 50911 and CGP 46381, but not by the GHB receptor antagonist, NCS-382. Thus, the authors concluded that the sedative effects of 1,4-BD were mediated by GABA_B_, but not by the GHB receptor [24].

In summary, the co-administration of GHB and ethanol induced strong toxicodynamic interactions probably due to the combined action of the GABA receptors, while toxicokinetic changes of GHB were not significant in animal studies. Since ethanol inhibits the conversion of 1,4-BD to GHB, the concentration of 1,4-BD in the body is increased by the co-administration of GHB with ethanol. The toxicodynamic changes from ethanol co-ingestion could vary depending on the use of GHB or its precursors.

**Table 3:** Animal studies on toxicokinetic and toxicodynamic changes in gamma-hydroxybutyric acid (GHB) following co-administration of ethanol.

Subject	Method		Result			Ref.
Male Wistar rats	Group	Saline, GHB (GBL), EtOH, GBL/EtOH	Toxicokinetics	GHB/EtOH → V_max_ (↓), V_T_ (↑), V_dss_ (↑)		[11]
	Protocol	Toxicokinetics: EtOH (infusion to steady-state EtOH target conc. 300–3000 µg/mL) followed by GHB (a single bolus, 400 mg/kg) Toxicodynamics: 20 min after target conc. of GHB (infusion to steady state GHB target conc. 200–1400 µg/mL) or EtOH (infusion to steady-state EtOH target conc. 1000–3000 µg/mL) Sedation test (RR): EtOH (3 g/kg, i.p.), GBL (0.3 g/kg, i.p.), GBL/EtOH	Toxicodynamics	RR: GHB/EtOH (>2000 µg/mL) → synergy; GHB/EtOH (lower conc.) → additivity		
				SR: GHB/EtOH (<1000 µg/mL) → antagonism; GHB/EtOH (higher conc.) → additivity		
				TC: GHB/EtOH (all conc.) → antagonism		
			Sedation (RR)	GBL/EtOH > EtOH > GBL		
Male Swiss-Webster mice	Group	Vehicle, GHB, EtOH, GHB/EtOH	Locomotor activity	Vehicle, EtOH > GHB > GHB/EtOH		[18]
	Protocol	Behavior test: GHB (0.1–1.0 g/kg, i.g.), EtOH (2.0–5.0 g/kg, i.g.)				
			RR	Vehicle, GHB, EtOH < GHB/EtOH		
			FGS	Vehicle > GHB, EtOH > GHB/EtOH		
			ISP	Vehicle > GHB, EtOH > GHB/EtOH		
			HS	Vehicle < GHB, EtOH < GHB/EtOH		
			Body temperature	Vehicle > GHB, EtOH > GHB/EtOH		
Male SD rats	Group	GHB, GHB/EtOH, GHB/EtOH/AZD or ARC	Toxicokinetics	GHB ≃ GHB/EtOH (oral) GHB/EtOH (i.v.) → terminal T_1/2_ (↑)		[19]
	Protocol	EtOH (2 g/kg, i.v.), GHB (0.6 g/kg, i.v. or 1.5 g/kg, i.g.), AR-C 155858 (MCT1 inhibitors, 5 mg/kg, i.v.), AZD-3965 (MCT1 inhibitors, 5 mg/kg, i.v.)				
			Sedation	EtOH < GHB/EtOH/AZD-3965 ≃ GHB < GHB/EtOH		
			MCT1 inhibition by AR-C 155858	GHB/EtOH → respiratory depression (↓) GHB/EtOH → CL_NR_ (↓) GHB → T_1/2_ (↑), CL_NR_ (↓), V_ss_ (↓)		
			MCT1 inhibition by AZD-3965	GHB conc. in brain and brain-to-plasma ratio at RRR (↓) GHB/EtOH → respiratory depression (↓) GHB/EtOH → CL_R_ (↑), V_ss_/F (↑), C_max_ (↓) GHB → CL/F (↑), CL_NR_/F (↑), V_ss_/F (↑), CL_R_ (↑), AUC (↓), C_max_ (↓), T_max_ (↓)		
Male SD rats	Group	GHB, EtOH, GHB/EtOH, GHB/EtOH/inhibitors or antagonists	Toxicokinetics	GHB ≃ GHB/0.1–0.4% EtOH		[20]
	Protocol	Sedation: EtOH (2.0 g/kg, i.v.), GHB (600 mg/kg, i.v.), L-lactate (MCT inhibitor, 66 mg/kg + 302.5 mg/kg/h), Bicuculline (bic, GABA_A_R antagonist, 1 mg/kg), SGS742 (SGS, GABA_B_R antagonist, 500, 1000 mg/kg), SCH50911 (SCH, GABA_B_R antagonist, 100, 200 mg/kg) Respiratory depression/fatality and toxicokinetics: GHB (600, 1500 mg/kg, i.v.), GHB/EtOH (steady-state conc. 0.1–0.2% or 0.3–0.4%), GHB/EtOH (steady-state conc. 0.1–0.2% or 0.3–0.4%)/inhibitors or antagonists Oral toxicokinetics:GHB (1.5 g/kg, i.g.), EtOH (2.5 g/kg, i.g.)	RR	GHB/EtOH > GHB, GHB/EtOH/L-lactate		
			Sleep time	GHB/EtOH≃GHB/EtOH/bic > GHB > GHB/EtOH/SGS or SCH > EtOH		
			GHB conc. in brain and brain-to-plasma ratio at RRR	GHB ≃ GHB/EtOH > GHB/EtOH/L-lactate		
			Respiratory Depression	Frequency:	GHB/EtOH/SCH → completely prevented GHB ≃ GHB/EtOH ≃ GHB/EtOH/bic	
				Tidal volume:	GHB/EtOH/SCH → completely prevented GHB > GHB/EtOH ≃ GHB/EtOH/bic	
			Fatality:	GHB/EtOH > GHB/EtOH/L-lactate > GHB, GHB/EtOH/SCH		
Male SD rats	Group	1,4-BD, GHB, EtOH, 1,4-BD/EtOH, GHB/EtOH	Mutual metabolic inhibition	EtOH/1,4-BD → significant; EtOH/GHB → not significant		[21]
	Protocol	Toxicokinetics: 1,4-BD (1.58, 6.34 mmol/kg, i.v. or oral), GHB (1.58, 1.79, 6.34 mmol/kg, i.v.), EtOH (6.34, 12.7 mmol/kg, i.v.) LRR test: 6.34 mmol/kg (i.v.)				
			Oral absorption of 1,4-BD	Rapid and complete		
			Total duration of LRR	1,4-BD > 1,4-BD/EtOH > GHB/EtOH > GHB		
Rhesus monkeys	Group	EtOH, GHB/EtOH	Reinforcing effects in self-administration	EtOH ≃ GHB/EtOH		[22]
	Protocol	Self-administration: EtOH (50, 100, 200 mg/kg/inj, i.v.), GHB (1.0, 3.2 mg/kg/inj, i.v.)	Demand functions in self-administration	EtOH ≃ GHB/EtOH		

AUC, area under the plasma concentration-time curve; C_max_, maximal concentration; CL, total clearance; CL_R_, renal clearance; CL_NR_, nonrenal clearance; conc., concentration; EtOH, ethanol; F, bioavailability; FGS, Forelimb grip strength; GBL, gamma-butyrolactone; GHB, gamma-hydroxybutyric acid; GABA_B_R, GABA_B_ receptor; GABA_A_R, GABA_A_ receptor; HS, Hindlimb splay; i.p., intraperitoneal injection; i.v., intravenous injection; i.g., intragastric administration; inj., injection; ISP, Inverted screen performance; LRR, loss of righting reflex; MCT, monocarboxylate transporter; RR, righting reflex; RRR, return of righting reflex; SD, Sprague-Dawley; SR, startle reflex to a hand clap; TC, tail clamp reaction; T_max_, time of maximal concentration; T_1/2_, half-life; V_max_, maximal metabolic rate; V_T_, peripheral volume of distribution; V_dss_, steady-state volume of distribution; V_ss_, steady-state volume of distribution; 1,4-BD, 1,4-butanediol; ≃, no significantly different; >, significantly different; <, significantly different; /, co-ingestion; ↓, decrease; ↑, increase.

**Table 4:** Animal and in vitro studies on toxicokinetic and toxicodynamic changes in 1,4-butanediol following co-administration of ethanol.

Subject	Method		Result			Ref.
Male LE rats (behavioral study), Male SD rats cardiovascular study)	Group	Saline, 1,4-BD, EtOH, 1,4-BD/EtOH	Behavioral study (response rate, % of control)	1,4-BD, EtOH → dose-dependently decrease, 1,4-BD/EtOH > 1,4-BD		[23]
	Protocol	Behavioral study (fixed-ratio 20 schedule of food presentation): EtOH (0.25–2 g/kg), 1,4-BD (0.18–0.56 g/kg) Cardiovascular study: Saline (1.0 mL, i.p. or i.v.), EtOH (2.0 g/kg, i.p.), 1,4-BD (0.18–1.0 g/kg, i.p. or i.v.), 1,4-BD (0.56 g/kg, i.v.)/EtOH (2.0 g/kg, i.p.)				
			Mean arterial blood pressure	1,4-BD > Saline 1,4-BD > 1,4-BD/EtOH		
			Heart rate	1,4-BD > Saline EtOH > Saline 1,4-BD > 1,4-BD/EtOH		
Male DBA/2JIco mice	Group	1,4-BD, GHB, EtOH/1,4-BD, 1,4-BD/inhibitors,1,4-BD/antagonists	LRR	4MP/1,4-BD, EtOH/1,4-BD, DS/1,4-BD < 1,4-BD NCS-382/1,4-BD ≃ 1,4-BD SCH50911/1,4-BD < 1,4-BD CGP 46381/1,4-BD < 1,4-BD		[24]
	Protocol	GHB and 1,4-BD (0.2–1 g/kg, i.p.), EtOH (1 g/kg, i.p.), 4-methylpyrazole (4MP, ADH inhibitor, 0.1 mg/kg, i.p.), disulfiram (DS, ALDH inhibitor, 1–30 mg/kg, i.p.), NCS-382 (GHB receptor antagonist, 0.25 g/kg, i.p.), SCH50911, CGP46381 (GABA_B_ receptor antagonist, 0.1 g/kg, i.p.)				
Male SD rats	Group	Saline, GHB, 1,4-BD, EtOH/1,4-BD	Mean arterial blood pressure	1,4-BD > GHB		[25]
	Protocol	Cardiovascular study: GHB (0.56–10 g/kg, i.g.), 1,4-BD (0.18–1.0 g/kg, i.g.), 1,4-BD (1.8 g/kg, i.g.) Mortality: EtOH (2.0 g/kg, i.p.)/1,4-BD (1.8 g/kg, i.g.)	Heart rate	1,4-BD > GHB		
			Mortality	1,4-BD > EtOH/1,4-BD		
Male SD rats	Group	Vehicle, EtOH, 1,4-BD, 1,4-BD/EtOH, GBL	Electroencephalogram activity	1,4-BD > GBL, EtOH followed by 1,4-BD < GBL		[26]
	Protocol	1,4-BD (1 g/kg, i.g.), EtOH (3 g/kg, i.g.), GBL (400 mg/kg, i.g.)	LRR	EtOH < EtOH/1,4-BD		
			GHB conc. in brain and liver	1,4-BD > EtOH/1,4-BD		
			EtOH conc. (blood)	EtOH ≃ EtOH/1,4-BD		
Male SD rats (in vitro)	Group	1,4-BD, EtOH, Pyrazole, Disulfiram	Conversion rate of 1,4-BD to GHB	EtOH in brain and liver (↓) Pyrazole, Disulfiram in liver (↓)		[27]
	Protocol	Conversion rate of 1,4-BD to GHB in brain and liver and oxidation rate of 1,4-BD to GHB: 1,4-BD (8 mM), EtOH (10, 20 mM), pyrazole (ADH inhibitor, 1 mM), disulfiram (ALDH inhibitor, 1 mM)				
			Oxidation of 1,4-BD to GHB	Competitively inhibited by EtOH		
Human livers (autopsy within 72 h after death)	Group	1,4-BD, 1,4-BD/EtOH, 1,4-BD/AL, 1,4-BD/inhibitors	Conversion of 1,4-BD to GHB	1,4-BD/EtOH (↓), 1,4-BD/AL (↑)		[28]
	Protocol	10 human livers (5 men, 5 women, 43–79 years old) Conversion of 1,4-BD to GHB: 1,4-BD (3–80 mM), EtOH (0–2 mM), acetaldehyde (AL, ADH inhibitor, 0–2 mM) Inhibitors efficiency: 1,4-BD (0.5–5 mM) + ADH inhibitors (fomepizole, pyrazole) or ALDH inhibitors (disulfiram, cimetidine)	Inhibitors efficiency	Fomepizole:	GHB formation (↓), most potent inhibitor	
				Pyrazole:	GHB formation (↓)	
				Disulfiram:	GHB formation (↓)	
				Cimetidine:	GHB formation (↓), weakest inhibitor	
Male SD rats	Group	1,4-BD, EtOH, 1,4-BD/EtOH	EtOH conc. (blood)	EtOH ≃ 1,4-BD/EtOH		[29]
	Protocol	Measurement of EtOH and 1,4-BD levels, mortality rate and histochemistry (brain. Liver, kidney): 1,4-BD (1 g/kg, i.g.), EtOH (3 g/kg, i.p.)	1,4-BD conc. (brain, liver, kidney)	1,4-BD/EtOH > 1,4-BD		
			Mortality rate	1,4-BD/EtOH > 1,4-BD		
			Histological alterations	EtOH → no change; 1,4-BD → hyperemia in all organs (↑); 1,4-BD/EtOH → tissue damage (↑), fatty infiltration and necrosis in liver, extensive medullary necrosis in kidney)		

ADH, alcohol dehydrogenase; AL, acetaldehyde; ALDH, aldehyde dehydrogenase; conc., concentration; EtOH, ethanol; GBL, gamma-butyrolactone; GHB, gamma-hydroxybutyric acid; i.p., intraperitoneal injection; i.v., intravenous injection; i.g., intragastric administration; LE, Long–Evans; LRR, loss-of-righting reflex; SD, Sprague-Dawley; 1,4-BD, 1,4-butanediol; ≃, no significantly different; >, significantly different; <, significantly different; /, co-ingestion; ↓, decrease; ↑, increase.

## 8. Drug Discrimination or Responding Following GHB and Ethanol Co-Administration

The effects of the co-ingestion of GHB and ethanol on drug discrimination or response in animals are summarized in Table 5. Drug discrimination is a series of procedures found to be particularly useful for characterizing similarities and differences among drugs and their abuse liability [94,95,96,97]. Cook et al. reported that ethanol produced less than 50% of GHB-like discriminative stimulus effects, but GHB did not affect the GHB-like discriminative stimulus effects of ethanol [18]. Metcalf et al. found that GHB and ethanol were not cross-generalized in the ethanol- and GHB-trained rats, respectively. However, there was a synergistic interaction between GHB and ethanol when administered as a mixture in the GHB- and ethanol-trained rats. When the GHB- and ethanol-trained rats were tested with the mixed dose of 225 mg/kg GHB and 750 mg/kg ethanol, complete generalization was produced in both groups [30]. Regarding the response in rats under a fixed-ratio (FR) 10 schedule of sugar solution presentation, GHB decreased the drug response rate in a dose-dependent manner, and the co-administration of GHB and ethanol further decreased the response rate. However, the GHB antagonist, NCS 382, did not appear to interfere with the effects of GHB. The authors of this study suggest that many of the behavioral actions induced by exogenous GHB are due to GHB action at sites other than the GHB receptor [31]. The effects of reinforcement types, such as food or water, and administration routes, such as intraperitoneal (IP) or intragastric (IG) administration on stimulus generalization, were also studied. Food maintained significantly higher response rates than water. The IG-water group was most sensitive to a lower dose of GHB, and only the IP-water group failed to generalize to orally-administered GHB. GBL and 1,4-BD were fully substituted, except for in the IP-food group, while ethanol was partially substituted in all groups. The combination of GHB (150 mg/kg) and ethanol did not show any additive effects in all groups [32]. The finding that animals can partially or fully generalize among GHB, ethanol, and their mixture is further evidence that the effects of GHB and ethanol are not completely identical but may be involved in each different subtype of GABA receptors for enhanced inhibitory effects.

## 9. Future Directions and Conclusions

**Figure 2:**
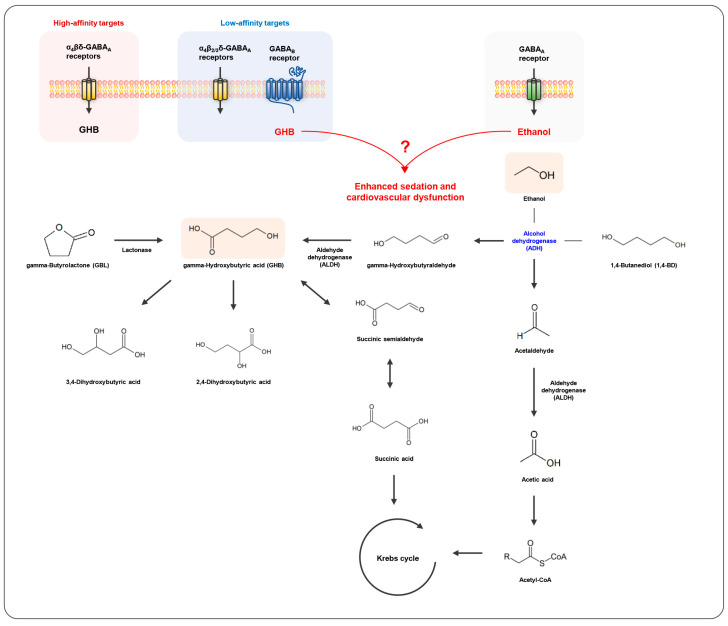
Interactions between gamma-hydroxybutyric acid (GHB) and ethanol.

Clinical cases reported that the combined exposure to GHB and ethanol can cause severe sedation and respiratory depression, potentially resulting in death more frequently, compared with the administration of GHB alone. Previous animal studies also demonstrated that the combined administration of GHB and ethanol enhanced sedation and cardiovascular dysfunction. However, the toxicodynamic alterations caused by the co-administration of GHB and ethanol poorly correlate with toxicokinetic changes in GHB. Both GHB and ethanol are metabolized to inactive metabolites through the Krebs cycle. Since the toxicological effects of both GHB and ethanol are mainly caused by the action of the GABA receptors, i.e., the GABA_B_ and the GABA_A_ receptors, respectively, it is assumed that the enhanced inhibitory effects of GHB caused by ethanol could be more or less additive. 1,4-BD is rapidly and extensively converted to GHB, and has stronger and more prolonged sedative effects than GHB [24,98]. However, the co-administration of 1,4-BD with ethanol results in a decrease in the sedative effects since 1,4-BD is converted to GHB by ADH, where ethanol acts as an inhibitor (Figure 2). There is still insufficient evidence to understand the action mechanism of the interactions between GHB or its precursors and ethanol, which could be an obstacle for the diagnosis and treatment of GHB intoxication in the future. Therefore, toxicokinetic investigations of not only GHB but also other metabolites related with GHB and ethanol, as well as toxicodynamic interactions, need to be performed using a variety of exposure doses or durations in animal and clinical settings.

## Figures and Tables

**Table 1:** Clinical case studies of gamma-hydroxybutyric acid (GHB)/gamma-butyrolactone (GBL) and ethanol-related intoxication.

Subject	Method		Result		Ref.
Patients (ED)	Subject Period Country Group	710 of 5629 patients October 2013–September 2014 10 European countries A (GHB or GBL) B ([GHB or GBL]/other drugs)	Patient’s characteristics	Mean age (31 years), male (n = 592), female (n = 118) Group A (n = 201), Group B (n = 509) Other drugs consumed with GHB/GBL: EtOH (50%) > amphetamine derivatives (36%) > cocaine (12%) > cannabis (8%)	[12]
			Common clinical features (Group A + B, % of patients)	Altered behavior (39%), reduced consciousness (34%), anxiety (14%)	
			Specific symptoms (Group A vs. Group B, % of patients)	Vomiting (3% vs. 15%), cardiovascular symptoms (1.5% vs. 5.3%)	
Patients (ED)	Subject Period Country Group	609 of 17,371 patients October 2013–December 2016 14 European countries A (GHB or GBL) B ([GHB or GBL]/EtOH)	Patient’s characteristics	Mean age (32 years), male (n = 493), female (n = 116) Group A (n = 183), Group B (n = 426)	[13]
			Common clinical features (Group A + B, % of patients)	Decreased consciousness (56.1%), agitation or aggressive behavior (33.6%)	
			Specific symptoms (Group A vs. Group B, % of patients)	Decreased consciousness (49.1% vs. 58.9%), bradycardia (23.5% vs. 15.7%)	
Patients (ED)	Subject Duration Country Group	48 patients (65 episodes) January 2001–December 2003 European countries A (GHB or GBL) B ([GHB or GBL]/EtOH	Patient’s characteristics	Mean age (24 years), male (n = 31), female (n = 17) Group B (48% of episodes)	[14]
			Common clinical features (Group A + B, % of episodes)	Bradycardia (38%), hypotension (6%), hypothermia (48%)	
			Specific symptoms (Group A + B vs. Group B, % of episodes)	Agitation (17% vs. 29%), vomiting (31% vs. 39%)	

ED, emergency department; EtOH, ethanol; GBL, gamma-butyrolactone; GHB, gamma-hydroxybutyric acid; /, co-ingestion.

**Table 2:** Human studies on toxicokinetic and toxicodynamic changes following gamma-hydroxybutyric acid (GHB) and ethanol co-administration.

Subject	Method		Results			Ref.
Healthy adults	Subject	16 healthy adults 7 men, 9 women, 22–34 years old 7 Whites, 6 Asian/Pacific Islanders, 1 Latino, 2 multiple ethnicities	Toxicokinetics	GHB:	GHB ≃ GHB/EtOH	[15]
				EtOH:	EtOH ≃ GHB/EtOH	
			Toxicodynamics	O_2_sat.:	Placebo > GHB ≃ EtOH > GHB/EtOH	
	Protocol	Randomized, double-blinded, crossover design 50 mg/kg GHB (Xyrem®), 0.6 g/kg EtOH		BP:	Placebo ≃ GHB > GHB/EtOH ≃ EtOH	
				HR:	EtOH ≃ GHB/EtOH > placebo ≃ GHB	
				Skin temp.:	GHB, EtOH, GHB/EtOH > placebo	
			Adverse events	GHB/EtOH (2 hypotension, 6 vomiting)		
Healthy adults	Subject	16 healthy adults 7 men, 9 women, 22–34 years old 7 Whites, 6 Asian/Pacific Islanders, 1 Latino, 2 multiple ethnicities	Urinary GHB conc.	GHB/EtOH < GHB (0–3 h)		[16]
			Renal clearance	GHB ≃ GHB/EtOH		
	Protocol	Randomized, double-blinded, crossover design 50 mg/kg GHB (Xyrem®), 0.6 g/kg EtOH				
Healthy adults	Subject	24 healthy adults 12 men, 12 women, 18–43 years old 91.7% Caucasian, 4.2% Asian, 4.2% others	Toxicokinetics	No interaction between SMO.IR and EtOH		[17]
			Toxicodynamics	Physiological parameters: no effect at both 60 and 165 min after administration of SMO.IR/EtOH Subjective parameters (within 60 min after administration of SMO.IR/EtOH): alertness and stimulation (↑), sedation (↓)		
	Protocol	Randomized, double-blinded, crossover design 2.25 g SMO.IR, 0.7 g/kg EtOH (male), 0.57 g/kg EtOH (female)				
			Vital signs and physical examinations	All normal		

BP, blood pressure; conc., concentration; EtOH, ethanol; GHB, gamma-hydroxybutyric acid; HR, heart rate; O_2_sat, Oxygen saturation; skin temp., skin temperature; SMO.IR, solid immediate release formulation of sodium oxybate; ≃, no significantly different; >, significantly different; <, significantly different;/, co-ingestion; ↓, decrease; ↑, increase.7. Toxicokinetic and toxicodynamic interactions between GHB and ethanol in animals.

**Table 5:** Drug discrimination or response following gamma-hydroxybutyric acid (GHB) and ethanol co-administration.

Subject	Method		Result		Ref.
Male Swiss-Webster mice	Group	Vehicle, GHB, EtOH, GHB/EtOH	Drug discrimination:	EtOH: <50% GHB-like discriminative stimulus effects GHB: no alteration in the GHB-like discriminative stimulus effects of EtOH	[18]
	Protocol	Discrimination training under FR 10 schedule of sweetened condensed milk presentation: GHB (0.1 g/kg, s.c.) and water Discrimination testing: GHB (0.1 g/kg, s.c.), EtOH (1–2.5 g/kg, i.p.), NCS382 (GHB antagonist, 0.03–0.1 g/kg, i.p.)			
Male LE rats	Group	GHB trained, EtOH trained, GHB/EtOH trained	Generalization	GHB trained: GHB (225 mg/kg)/EtOH (750 mg/kg) → full generalization, higher mixture doses → >71% generalization EtOH trained: GHB (>225 mg/kg)/EtOH (>750 mg/kg) → full generalization GHB/EtOH trained: GHB (>225 mg/kg)/EtOH (>750 mg/kg) → full generalization	[30]
	Protocol	Discrimination training under FR 10 schedule of food presentation: GHB (300 mg/kg, i.g.), EtOH (1 g/kg, i.g.), GHB (150 mg/kg, i.g.)/EtOH (500 mg/kg. i.g.) Discrimination/generalization testing: GHB (75–900 mg/kg), EtOH (250–3000 mg/kg), GHB/EtOH (a combination of one half of each of the respective doses of GHB and EtOH)			
			Response rates	GHB trained: GHB/EtOH > GHB ≃ EtOH EtOH trained: GHB/EtOH ≃ EtOH > GHB GHB/EtOH trained: GHB/EtOH ≃ EtOH > GHB	
Male Lewis rats	Group	Vehicle, GHB, EtOH, GHB/EtOH, GHB/NCS382	Drug responding	GHB: dose-relatedly decreased GHB/EtOH: greatly decreased GHB/NCS382: not antagonize the rate-decreasing effects of GHB	[31]
	Protocol	Drug responding of rats under FR 10 schedule of sugar solution presentation: GHB (180, 300 mg/kg, i.p.), EtOH (0.1–0.8 g/kg, i.p.), NCS382 (GHB antagonist, 3.2, 32.0 mL/kg, i.p.)			
Male SD rats	Group	IP-food, IG-food, IP-water, IG-water	GHB dose-response function	IG-food ≃ IP-food > IG-water ≃ IP-water	[32]
	Protocol	Discrimination training under FR 10 schedule of food or water presentation: GHB (300 mg/kg) and vehicle GHB dose-response function: GHB (1 mL/kg, i.p.), GHB (10 mL/kg, i.g.) Stimulus generalization test: GHB (75–300 mg/kg, i.p. and i.g.), GHB (400 mg/kg, i.g.), GBL (50–200 mg/kg, i.p.), 1,4-BD (100–400 mg/kg, i.p.), EtOH (1–3 g/kg, i.g.), GHB (150 mg/kg, i.g.)/EtOH (1–2 g/kg, i.g.)	Stimulus generalization:	GHB (i.p.): IG-water > IP-water ≃ IG-food ≃ IP-food GHB (i.g.): IP-water < IG-water ≃ IG-food ≃ IP-food GBL, 1,4-BD: fully substituted for GHB (except for in IP-Food) EtOH: partially substituted in all groups GHB/EtOH: no additive in all groups	

EtOH, ethanol; FR, fixed-ratio; GBL, gamma-butyrolactone; GHB, gamma-hydroxybutyric acid; i.p., intraperitoneal injection; i.g., intragastric administration; LE, Long–Evans; SD, Sprague-Dawley; s.c., subcutaneous injection; 1,4-BD, 1,4-butanediol; ≃, no significantly different; >, significantly different; <, significantly different; /, co-ingestion.
